# A check list and population trends of invasive amphibians and reptiles in Taiwan

**DOI:** 10.3897/zookeys.829.27535

**Published:** 2019-03-11

**Authors:** Ko-Huan Lee, Tien-Hsi Chen, Gaus Shang, Simon Clulow, Yi-Ju Yang, Si-Min Lin

**Affiliations:** 1 School of Life Science, National Taiwan Normal University, Taipei 116, Taiwan; 2 Department of Biological Sciences, Macquarie University, Sydney, NSW, Australia; 3 Institute of Wildlife Conservation, National Pingtung University of Science and Technology, Pingtung 912, Taiwan; 4 Department of Biotechnology, Ming Chuan University, Taoyuan 333, Taiwan; 5 Department of Natural Resources and Environmental Studies, National Dong Hwa University, Hualien 974, Taiwan

**Keywords:** Alien species, CITES, fauna checklist, international trade, island biogeography, IUCN

## Abstract

Invasive species have impacted biodiversity all around the world. Among various ecosystems, islands are most vulnerable to these impacts due to their high ratio of endemism, highly specialized adaptation, and isolated and unique fauna. As with other subtropical islands, Taiwan faces constant risk of biological invasions and is currently ranked as one of the countries most affected by invasive amphibians and reptiles. In this paper, a comprehensive checklist of all known exotic amphibians and reptiles is provided, including twelve species which have successfully colonized Taiwan and six species with a controversial status. We provide an update on the knowledge of all these species including their distribution, colonization history, threats to native animals, and population trends based on literature records, fauna surveys, and data collected during invasive species eradication and control programs. A list of species with high invasive potentials is also provided. This study reports, for the first time, a comprehensive survey of invasive herpetofauna in Taiwan, which should provide a valuable reference to other regions which might suffer from similar invasion risk.

## Introduction

Invasive species have been listed as one of the major threats to global biodiversity (Charles and Dukes 2008, Bellard et al. 2016, Early et al. 2016). The negative impacts of invasive species include predation, competition, hybridization, and introduction of exotic pathogens (Mooney and Cleland 2001, Gurevitch and Padilla 2004, Hulme 2014, Doherty et al. 2016). These in turn have contributed to the decrease of global diversity either by directly eliminating native species (decrease of alpha diversity), or by indirectly reducing the local uniqueness because of homogenization (decrease of beta diversity). Among various ecosystems, islands are especially sensitive to the impacts of invasive species (O’Dowd et al. 2003, Sax and Gaines 2008). Insular species are usually kept isolated from their mainland relatives, adapt to specific niches on the islands, and represent a high ratio of endemism by being distributed in a comparatively narrow range. These species are considered at greatest risk from biological invasions, and explains in part why a majority of human-induced extinction has occurred on islands during the last several centuries (Fritts and Rodda 1998, Blackburn et al. 2004, Wyatt et al. 2008).

Taiwan is a medium-sized island located approximately 130 km east from continental Asia. Located at the border between the Palearctic and Indomalaya regions, fauna on this island consists of evolutionary lineages from both of these biogeographic regions (Toda et al. 1998, Shih et al. 2006, Yu et al. 2014, Tseng et al. 2018). Due to low oversea dispersal ability, herpetofauna represents the highest proportion of endemism among terrestrial vertebrates in Taiwan (Zhang-Jian 2002). Excluding marine species (sea turtles and sea snakes), there are 37 amphibians and 85 reptiles native to the island (Shang et al. 2009), with new species still being discovered in recent years (You et al. 2015, Wu et al. 2016, Wang et al. 2017). Among these, five salamanders (100%), 14 frogs (44%), 18 lizards (55%), and 14 snakes (29%) are endemic to the island (several of these are endemic subspecies). In addition to the high levels of endemism, amphibians and reptiles in Taiwan are also characterized by their remarkable fine-scaled differentiation. Phylogenetic studies have indicated several in situ speciation cases within the limited range of this island, while there are several cases of restricted geographic distribution between sibling taxa (Lai and Lue 2008, Lin et al. 2012, Tseng et al. 2015). Most endemic species on the island occupy only a small distribution, while the contact zone(s) between sibling species provide a valuable laboratory for evolutionary studies (Tseng et al. 2014, Wang et al. 2017).

Like other islands, Taiwan has suffered from biological invasions. Harbors in Taiwan have long played the role of international transfer stations for trade among adjacent regions; a considerable proportion of trade materials includes agricultural products, fishery products, garden plants, live animals, and wildlife products. Furthermore, keeping amphibians and reptiles as pets has become more popular in recent years. Based on a global review of invasive herpetofauna around the world by Capinha et al. (2017), it was estimated there are now 10 exotic species of herpetofauna in Taiwan, ranking it as the 10^th^ most invaded country by herpetofauna in the world. However, more recent surveys by the authors of this paper have identified several other species of invasive herpetofauna, thus creating a need for a more up to date review of the invasive herpetofauna present in Taiwan and the threats that these species pose. Moreover, we also aim to improve the current status that a large proportion of information of these invasive species are based merely on folk information, and has never been formally published.

In this paper, we provide an up to date and detailed checklist of exotic amphibian and reptile species which have successfully colonized Taiwan. For each species, we collected information on their colonization history, the potential threats they pose to local species and ecosystems, eradication and control attempts conducted by scientists, and some new data collected during these attempts. Finally, we made some broad assumptions on their future trends based upon observations and data collected in field. We hope this will provide a valuable reference for conservation managers both in Taiwan and in other regions that face similar invasion risks.

## Materials and methods

We collected all available information on invasive amphibians and reptiles in Taiwan (names and authorities provided in Table 1), including a thorough search of the literature, and data collected during fauna surveys from several ongoing invasive species eradications, control, and research programs. Information of species listed below was based on some of the authors’ research outcomes: *Polypedatesmegacephalus*, *Trachemysscriptaelegans*, *Physignathuscocincinus*, *Chamaeleocalyptratus*, *Iguanaiguana*, *Anolissagrei*, *Gekkogecko*, and *Geckomonarchus*. Four other known invasive species were not studied directly by us; instead we collected available information since 1980s. These species include *Kaloulapulchra*, *Fejervaryacancrivora*, *Hemidactylusbrookii*, and *Eutropismultifasciata*.

In addition to the above commonly-recognized invasive species, recent studies have provided evidence to suggest that several long-occurring reptile species traditionally considered native to Taiwan may have indeed been relatively recent invaders. These include *Mauremysreevesii*, *Hemidactylusfrenatus*, *Lepidodactyluslugubris*, *Hemiphyllodactylustypus*, and *Indotyphlopsbraminus*. The American Bullfrog *Lithobatescatesbeianus*, on the other hand, was traditionally thought to be an established invasive species, but there is considerable doubt as to whether they are actually self-sustaining or whether they are simply continually released. Collectively, these species are listed as having a “controversial status”, with relevant discussion below.

Finally, Taiwan is frequently exposed to accidental or intentional release of exotic animals that are not yet considered established and invasive. A large proportion of these animals constitute escaped or released pets. Although frequently reported by animal rescue centers, these species have not yet established breeding populations and are thus not considered invasive. We have categorized these species as “high-risk” that have a high likelihood of establishing as invasive in the future (names and authorities in Table 2), and have made brief comments on these in the last section.

## Results and discussion

Based on our review, we determined that there is a total of three amphibian and nine reptile species that have established stable, invasive populations in Taiwan (Table 1). Seven of these have been funded for eradication programs, of which one (*K.pulchra*) was ceased in recent years, and another (*E.multifasciata*) conducted only for the population on Green Island. *Chamaeleocalyptratus* and *Gekkogecko* were captured opportunistically by students, herpers, or pet keepers; the remaining species have never received official intervention actions.

**Table 1.** A list of invasive amphibians and reptiles in Taiwan.

**Species**	**1^st^ record**	**Possible origin**	**Removal fund source**	**Trend**
**Amphibians**				
*Kaloulapulchra* Gray, 1831; Banded Bullfrog	1997	Timber trades (?)	Government + NGO^1^	PE
*Fejervaryacancrivora* (Gravenhorst, 1829); Mangrove Frog	2005	Imported fish fry	None	PE
*Polypedatesmegacephalus* Hallowell, 1861; Spot-legged Tree Frog	2006	Horticultural plants	Government + NGO	PE
**Turtles**				
*Trachemysscriptaelegans* (Wied, 1838); Red-eared Slider	N/A	Intentional release	None	PE
** Squamata **				
*Physignathuscocincinus* Cuvier, 1829; Chinese Water Dragon	2010	Intentional release	Government + private	PE
*Chamaeleocalyptratus* Duméril and Bibron, 1851; Veiled Chameleon	2011	Intentional release	Private people	PP
*Iguanaiguana* (Linnaeus, 1758); Common Green Iguana	2004	Intentional release	Government	PE
*Anolissagrei* Dumeril and Bibron, 1837; Brown Anole	2000	Horticultural plants	Government + NGO	PE
*Gekkogecko* (Linnaeus 1758); Tokay Gecko	2008	Intentional release (?)	Private people	PP
*Geckomonarchus* (Schlegel, 1836); Spotted House Gecko	2009	International trades	Government	PE
*Hemidactylusbrookii* Gray, 1845; Brook’s House Gecko	2018	International trades	None	PE
*Eutropismultifasciata* (Kuhl, 1820); Many-lined Sun Skink	1992	Timber trades (?)	Government^2^	PE
**Species with a controversial status**				
*Lithobatescatesbeianus* (Shaw, 1802); American Bullfrog	N/A	Intentional release	None	?
*Mauremysreevesii* (Gray, 1831); Reeves’ Turtle	1934	Intentional release	None	PD
*Hemidactylusfrenatus* Dumeril and Bibron, 1836; Common House Gecko	1885	Unknown	None	PE
*Lepidodactyluslugubris* (Dumeril and Bibron, 1836); Morning Gecko	1984	Unknown	None	PP
*Hemiphyllodactylustypus* Bleeker, 1860; Indopacific Tree Gecko	1985	Unknown	None	PP
*Indotyphlopsbraminus* (Daudin, 1803); Brahminy Blindsnake	?	Unknown	None	PP

PE: population expansion; PP: population persistency; PD: population decline ^1^ – The governmental support has ceased for several years. ^2^ – Governmental support for removal only on Green Island.

We determined that one frog (*L.catesbeianus*), one turtle (*M.reevesii*), and four squamates should be listed as having a controversial invasion status. In the first case, there is no confirmed evidence that *L.catesbeianus* has established a stable breeding population in Taiwan. In contrast, *M.reevesii* and *H.frenatus* should be revised to be considered as introduced species due to new lines of evidence based on genetic data and historical records (not from this study), both of which are discussed below. The three parthenogenetic squamates, *L.lugubris*, *H.typus*, and *I.braminus*, are considered invasive in Taiwan according to some authors (Ota et al. 2004, Kraus 2009). These species suffer from data deficiency and require further studies to conclusively confirm their status in Taiwan.

In terms of population trends, *M.reevesii* seems to have experienced dramatic population declines in the late 20^th^ century and has become near-extinct, although the reasons for this are unknown. Several medium- to large-sized lizards (e.g., *C.calyptratus* and *G.gecko*) were successfully, albeit temporarily controlled by students and pet keepers, primarily due to their market value, which led to at least a temporary reduction in the population size. One invasive frog (*F.cancrivora*) appears to be stable in population size, while others have continued to increase in population size over time with no signs of plateauing.

The 14 species summarized in Table 2, considered high-risk to become invasive in Taiwan, are either common in pet shops, frequently escape and are found in near-urban regions, or experience high levels of accidental invasion by international traders. These species are considered likely to invade Taiwan if no biosecurity restriction policy is put in place in the near future.

In the following sections, we discuss the detailed information from all the invasive species in these lists.

**Table 2.** A list of species with released individuals being frequently discovered, or with high invasive potential.

Species	Frequency in pet trades^1^	Records of escaped individuals^2^
**Amphibians**		
*Cynopsorientalis* (David, 1873); Oriental Fire-bellied Newt	Very high	Medium
*Rhinellamarina* (Linnaeus, 1758); Cane Toad, Marine Toad	Medium	Low
*Polypedatesleucomystax* (Gravenhorst, 1829); White-lipped Treefrog	Low	Low
** Squamata **		
*Anoliscarolinensis* Voigt, 1832; Green Anole	Low	Low
*Salvatormerianae* (Dumeril & Bibron, 1839); Black-and-white Tegu	High	High
*Varanusniloticus* (Linnaeus, 1766); Nile Monitor	Medium	Medium
*Varanussalvator* (Laurenti, 1768); Common Water Monitor	Medium	Medium
*Malayopythonreticulatus* (Schneider, 1801); Reticulated Python	Medium	Medium
*Pythonbivittatus* Kuhl, 1820; Burmese Python^3^	Medium	Medium
**Turtles**		
*Macrochelystemminckii* Troost, 1835; Alligator Snapping Turtle	High	High
*Chelydraserpentina* (Linnaeus, 1758); Common Snapping Turtles	High	High
*Pseudemysconcinna* (Le Conte, 1830); Eastern River Cooter	Very high	Very high
*Trachemysscriptascripta* (Schoepff, 1792); Yellow-bellied Slider	Very high	Very high
**Crocodilians**		
*Caimancrocodilus* (Linnaeus 1758); Spectacled Caiman	Medium	Medium

^1^ – Definition of frequency in pet trades: very high: > 10 individuals in most pet stores; high: < 10 individuals in most pet stores; medium: < 10 in a low proportion of pet stores; low: occasionally available or none. ^2^ – Definition of escaped records: very high: frequently found in the wild; high: more than 5 records in urban or natural settings each year; medium: 1 – 5 records in urban or natural settings each year; low: occasional records or none. ^3^ – Population of *Pythonbivittatus* is native only in Kinmen Archipelago; individuals found in Taiwan are usually escaped pets.

### *Kaloulapulchra* (Gray, 1831)

**Figure 1.**
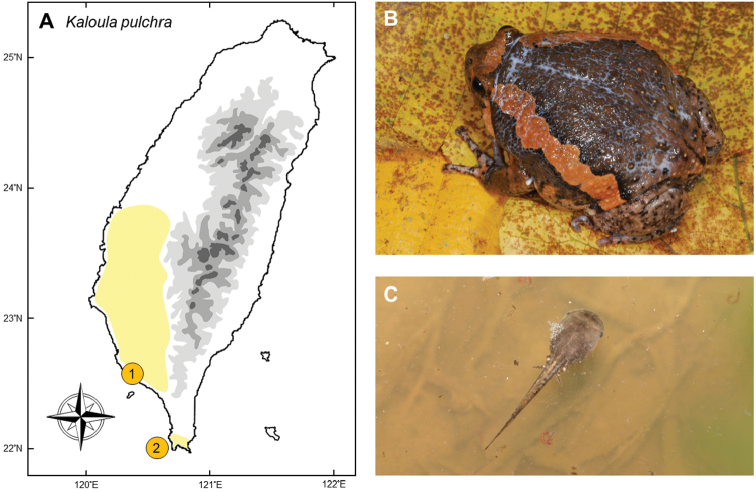
**A** The occurrence of *Kaloulapulchra* was first discovered in Kaohsiung (**1**), and later expanded northward to Yunlin, and southward to a disjunct location in Kenting (**2**) **B** the skin of this medium- to large-sized microhylid can secret toxins **C** their tadpoles are commonly found in the invasive regions. Photographed by Gaus Shang (**B**) and Yin-Hsun Yang (**C**).

**Natural distribution.** As a widely distributed species in South and Southeastern Asia, the west boarder of this medium-sized microhylid frog (Fig. 1) is India and Bangladesh, and it is widely distributed in Myanmar, Laos, Thailand, Malaysia, Singapore, certain areas of Indonesia, Cambodia, Vietnam, and south China, including Hong Kong and Macau (Vassilieva et al. 2016). It was introduced to Luzon, Philippines, probably through international pet trades (Diesmos et al. 2006). It was classified as “Least Concern” by IUCN Red List in 2004. Due to its wide distribution and tolerance of many kinds of habitats, it is not listed as threatened under any other legislative lists.

**Colonization history.** This species was first reported in 1997 by Yan-Hung Pan, from a military base in Linyuan District, Kaohsiung City (point 1 in Fig. 1A). In the following year, six specimens were captured at the same locality and sent to National Taiwan University for further identification. Based on morphology, one of us, Dr. Yi-Ju Yang, confirmed that this was the first record of *K.pulchra* in Taiwan. From the analysis of their mitochondrial genes, this species in Taiwan does not seem to have originated from nearby China (Lin 2007) and the origin of this frog remains unknown. Linyuan District is adjacent to Kaohsiung Harbor with many wood processing plants around this region and it is believed the species have entered via this route, as has been found to be the case in New Zealand (Gill et al. 2001).

The distribution and population size of this frog remained limited until the early 21^st^ century. The distribution started to increase after a significant flood in August 2009, which spread the frog to more lowland localities. In an investigation by Hou 2011, the species was found to have a disjunct distribution in Tainan, Kaohsiung and Pintung Counties. However, these isolated populations gradually expanded and merged, while newly established populations have occurred in Yunlin County and Kenting National park (point 2 in Fig. 1A); these two places represent the northern and southern-most boundaries of their current distribution up until 2017. New geographically isolated records of this frog were steadily being reported, such as Mudan Township in Pingtung County (Meng-Hsien Chuang, pers. comm.). To date, the hot spots for this species include Tainan (Gueiren, Guanmiao, and Longci), Kaoshiung (Lujhu, Tianliao, Tzukuan, Ciaotou, Daliao, Linyuan, and Kaohsiung Metropolitan Park), and Pintung (Neipu, Wanluan, Shinpi, and Kenting National Park) (Hou 2011, Chang 2012, Chen 2015).

**Threats to native species and ecosystems.** This species is usually abundant in invaded regions, but its threat to local fauna is still obscure. In Taiwan, *K.pulchra* usually shares similar food items with *Duttaphrynusmelanostictus* (Bufonidae) which preys heavily upon ants and other litter insects. Nevertheless, there is not yet clear evidence that the former represents strong competition with the latter (Liang 2005). Another consideration is that invasive poisonous amphibians can harm local naïve predators through lethal toxic ingestion (Fig. 1B; Burnett 1997, Letnic et al. 2008, Shine 2010). While the effect of their toxin on native predators remains unstudied, there are several toad-eating snakes that could potentially prey on *K.pulchra*, such as *Dinodonrufozonatum*, *Macropisthodonrudis*, *Rhabdophisformosanus*, and *Najaatra* (Karsen et al. 1998; Shang et al. 2009). Experiments on captive individuals of these snakes showed that at least *N.atra* can successfully consume it. A recent record in Tainan showed that *K.pulchra* may be sympatrically distributed with the critically endangered treefrog *Rhacophorusarvalis* (Rhacophoridae). The impact of *K.pulchra* on *R.arvalis*, which has an extremely narrow distribution, requires careful monitoring.

**Current status and trends.** The invasion dynamics of *K.pulchra* represented a typical trend of an invasive species: it remained in small numbers for quite a long period, and only started to expand after a “lag time” between initial colonization and the onset of rapid population growth and range expansion (e.g., Kowarik 1995, Sakai et al. 2001). Since the flood in 2009, its distribution and population size gradually increased. Although the speed of spread has been slow, its expansion continues to the present day. Because the species burrows under the soil (especially in the dry season), evaluation and removal of this long-lived frog is difficult. Typhoons and floods, as well as occasional release by pet keepers (Lin 2007), further facilitate their dispersal. The species has low mobility and thus eliminating it might be possible during the early stages of invasion; yet this phase seems to have passed in Taiwan and successful eradication seems unlikely.

The government initiated several programs to evaluate the distribution and population size of this species since 2005, but the programs did not persist (Hou 2011, Chang 2012). Although local government and nongovernment organizations have supported volunteers to remove this species, most of the captured individuals were adults and the number was too small to effectively decrease its population size (Old Bridge Association of Kaohsiung 2016). According to Hou 2011, at least 70% of eggs (tadpoles) and 30% of frogs must be removed in order to effectively decrease the population size. The ability to hibernate in mud and with a fair tolerance of overwintering in Taiwan, this species will likely gradually disperse northward (Chang 2012).

### *Fejervaryacancrivora* (Gravenhorst, 1829)

**Figure 2.**
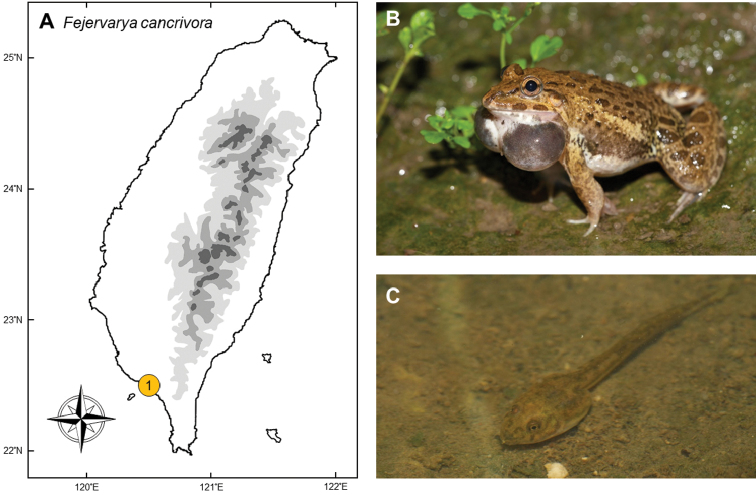
**A** Distribution of *Fejervaryacancrivora* is restricted to blackish water and fish ponds in Donggang, Linbian, Jiadong, and Fangliao townships of Pingtung County (**1**) **B** a male frog delivering the breeding call **C** a tadpole of *F.cancrivora*. Photographed by Yin-Hsun Yang.

**Natural distribution.** Inhabiting the coasts and mangroves across south Asia, this robust dicroglossid frog (Fig. 2) is known for its tolerance to brackish water in both larval and adult stages. The distribution ranges from the Philippines to Indonesia, as far east as Flores Island (Grismer 2011a). It also occurs in south China, Thailand, Peninsular Malaysia and west to India (Orissa and Pondicherry) (Grismer 2011a, Satheeshkumar 2011). This species has also been introduced to New Guinea and Guam (Menzies 1996, Christy et al. 2007a). The IUCN assessed this species as Least Concern, and it is not listed under CITES.

**Colonization history.** This species was first listed as present in Taiwan by Johnson TF Chen in his first (Chen 1956) and second (Chen 1969) editions of “A synopsis of the vertebrates of Taiwan”. However, no voucher specimen was mentioned in Chen’s records; and no further records were mentioned since Chen’s book. Based on the description in the books, it might have been a misidentification from the morphologically similar native species *Hoplobatrachusrugulosus*.

In June 2005, this frog was once again discovered by Mr Jia-Hui Lin, a teacher of Renhe primary school, Pingtung County. It was preliminarily identified by Dr Yi-Ju Yang and Cheng-En Li by photograph. Several specimens were later collected in July of the same year, and mating pairs and tadpoles were discovered in October. *Fejervaryacancrivora* was thus confirmed as a breeding population in southern Taiwan. This frog now has a restricted distribution in Taiwan to the river mouth of Donggang Stream and Linbian Stream, belonging to Donggang, Linbian, Jiadong, and Fangliao townships (point 1 in Fig. 2A).

Molecular analyses have shown that this population is closely related to the populations from Borneo, Sumatra, and the Malay Peninsula, but distantly related to adjacent populations in China and the Philippines (Kurniawan et al. 2010). Therefore, this population was confirmed to be exotic. Borneo, Sumatra, and the Malay Peninsula are famous for their aquaculture; the origin of this frog is considered likely to have been introduced with imported fry.

**Threats to native species and ecosystems.***Fejervaryacancrivora* normally utilizes brackish water, where almost no other amphibians exist. In Taiwan, they utilize fish farms, mangroves, and occasionally occur in orchards of wax apple, where local people use salty water to enhance the fertility of the plants. In inland areas, they sometimes occur in sympatry with native species *Fejervaryalimnocharis*, *Microhylafissipes*, and *Duttaphrynusmelanostictus*, but the population is not dominant. Owing to the low to medium abundance of the frog and the lack of relevant research, there is no evidence for competition between *F.cancrivora* and native species, nor for the effects on the native food-web through predation as well as by being preyed.

**Current status and trends.** This species is currently found only in Donggang, Linbian, Jiadong, and Fangliao townships of Pingtung County, and also in the mangroves of Dapeng Bay. The population is limited both in abundance and range, with no prominent sign of fast continuing spread. There has not been a proposal to conduct removal or research on this species.

### *Polypedatesmegacephalus* Hallowell, 1861

**Figure 3.**
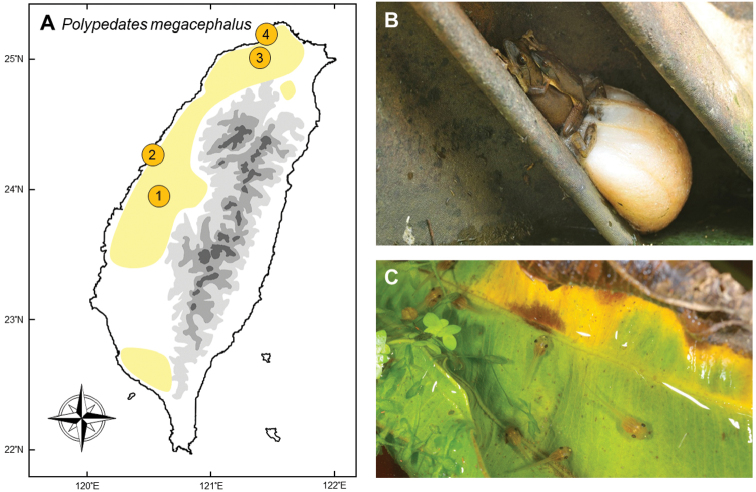
**A** Invasion of *Polypedatesmegacephalus* started in central Taiwan (Tienwei (**1**) and Wuchi (**2**)), and spread quickly by island hopping from habitat to habitat forward to northern Taiwan (Yingge (**3**) and Bali (**4)**) **B** a mating pair of adults with their foam nest **C** a small group of *P.megacephalus* tadpoles. Photographed by Yu-Jen Liang (**B**) and Gaus Shang (**C**).

**Natural distribution.** This medium-sized Old-world treefrog (Rhacophoridae, Fig. 3) is widespread in India, Bangladesh, Myanmar, Thailand, Laos, Cambodia, Vietnam and southern China, including Hainan Island (Fei et al. 2012, Vassilieva et al. 2016). It was reported to have invaded Guam from China through shipments (Christy et al. 2007a, Christy et al. 2007b). However, other brownish *Polypedates* spp. in East and Southeast Asia, such as *P.leucomystax* and *P.braueri*, form a group of morphologically similar species complex, which has caused taxonomic confusion in past decades. Therefore, the precise definition of members within this group needs to be clarified and might cause misidentifications (Kuraishi et al. 2013). The IUCN lists this species as Least Concern, and there are no trade restrictions.

**Colonization history.** This species was first recorded by a citizen of Wuchi, Taichung City who accidently brought a group of tadpoles home with aquatic plants from Tienwei, Changhua in 2006 (point 1 Fig. 3A). Upon metamorphosis, he realized this species might have not been recorded in Taiwan (Yang and Gong 2014). By 2010, there were established populations in Taichung (point 2), Taoyuan (point 3) and New Taipei City (point 4). The disjunct distribution implied that human-mediated dispersal had occurred, rather than motive expansion. It was suspected that this species was imported with aquatic plants, and was most likely to disperse through the nursery trade (Zhang 2008).

When this species was first found in 2006, it could only be found in Changhua and Taichung. During the first several years, this species formed a disjunct distribution in northern (Taipei and Taoyuan) and central (Taichung and Changhua) Taiwan. It expanded progressively to nearby regions, such as Keelung, Yilan, Hsinchu, Miaoli, Nantou, Yunlin, and Pingtung (Yang and Gong 2014, Chen 2015). In 2013, this species was found in one third of the surveyed areas (Yang et al. 2014); and expanded to 109 of 148 sampling sites (> 70%) in 2017 (Yang and Chen 2017).

**Threats to native species and ecosystems.***Polypedatesmegacephalus* preys primarily on small insects, and sometimes small vertebrates such as *Gekkohokouensis*, *Diplodermaswinhonis*, and *Microhylafissipes* (Chen 2014). The most serious threat to native fauna might be resource competition with local anura, especially *P.braueri* which occupies a similar niche. Compared to *P.braueri*, *P.megacephalus* is larger in body size and has a larger clutch size (Wu et al. 2010). Strong competitive exclusion has been documented when *P.megacephalus* invades into the habitat of *P.braueri*; among a number of localities which are apparently suitable to both species, only the former can now be found (Yang and Chen 2016, 2017).

**Current status and trends.** This species is still expanding rapidly, with individuals being able to migrate up to 744 meters in a single day (Chang 2016). In 2017, Yang and Chen (2017), reported that the species had invaded 13 of the 22 counties surveyed in 1,085 localities; most of which are disturbed areas such as parks and school campuses. They reported 13,225 individuals, making the species one of the most abundant amphibians on the island.

Monitoring and removal of this species began in 2011, supported by the Forestry Bureau. Hundreds of individuals were removed by volunteers every year from at least four hotspots: Bali (point 4 in Fig. 3A), Yingge (point 3), Taichung Metropolitan Park, and Tienwei (point 1) between 2012 and 2017 (Yang and Chen 2017). Removal projects have proven to effectively depress the population in these areas and facilitate other frogs to recover from local decline. Nevertheless, complete eradication is likely impossible by removal.

Current invasion patterns suggest the spread of this species will continue unabated. Management in the near future should focus on how the population size can be depressed and how to maintain the long term viability of native species. Current observations suggest this frog can utilize artificial water bodies and form large populations in disturbed areas. The removal of artificial water bodies could potentially reduce numbers of the frog without being harmful to native species. Ecological corridors between hot spots of this frog could be further interrupted by using fences in order to stop the expansion (Chang et al. 2013).

### *Trachemysscriptaelegans* (Wied, 1838)

**Figure 4.**
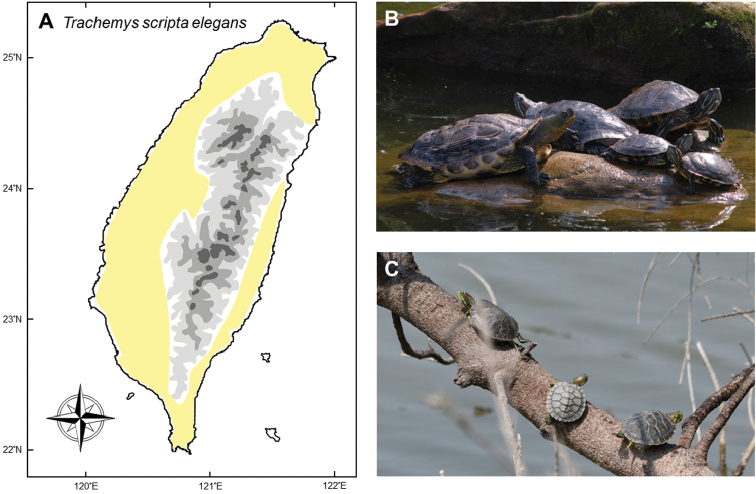
**A***Trachemysscriptaelegans* can be found in natural, semi-natural, or artificial wetlands in urban or suburban regions all around the island **B** they are usually found in sympatry with the native *Mauremyssinensis***C** the hatchling turtles show their potential to reproduce in some habitats. Photographed by Gaus Shang (**B**) and Yu-Jen Liang (**C**).

**Natural distribution.** This freshwater emydid turtle (Fig. 4) is originally distributed in wetlands of southern United States, from Iowa to Florida, and northern Mexico, including Coahuila, Nuevo Leon and Tamaulipas (Ernst 1990, IUCN, Reptile database). It has invaded areas outside of its native distribution in USA and many countries worldwide, such as Australia (Burgin 2006), China (Shi 2000), France (Cadi et al. 2004), Italy (Luisellie et al. 1997), Japan (Uchida 1989, Ota 1995), and New Zealand (Thomas and Hartnell 2000). The IUCN lists this species as one of the top 100 of the world’s worst invasive animals (Lowe et al. 2000). Turtle farms in the USA used to export millions of individuals every year to Korea, Japan, Thailand, and countries in South Africa and Europe, either for the pet trade or religious “Mercy Ceremony” (CITES Trade Database, Telecky 2001) and turtle farms bloomed in many Asian countries such as Thailand, Malaysia and China (Ramsay et al. 2007, Shi et al. 2008).

**Colonization history.** Invasion of this species can be traced to the late decades of 20^th^ Century through intentionally being released by pet owners and religious activities (Ling 1972, Chen 2006). Chen and Lue (1998) reported the first record of feral populations in the Keelung River, Taipei; although the real age of their invasion must be much earlier. A more comprehensive survey in 2006 showed that this species had been distributed across western Taiwan, mostly in the northern and central regions, and also on Kinmen Islands (Chen 2006).

Nowadays, this species can be found in many aquatic systems in Taiwan, especially artificial ponds and rivers close to urban areas. Because of the pet market, citizens can get this species very easily, resulting in a fast assisted dispersal rate. Moreover, this species is sold near temples where Buddhists buy animals for their mercy ceremonies, which further facilitate this species to establish new populations.

**Threats to native species and ecosystems.** This species likely occupies most suitable water bodies through human-mediated dispersal (Heidy Kikillus et al. 2010). It coexists with several other native chelonians (Chen 2006), such as *Mauremyssinensis* and *M.mutica* in Taiwan (Fig. 4B), and *M.reevesii* in Kinmen Island. Although evidence on direct competition is scarce, the threat to local ecosystems could be underestimated. First, it has been shown that *T.s.elegans* carried parasites which could switch hosts to *Mauremysleprosa* in northern Spain and southern France (Meyer et al. 2015). Second, this species is an opportunistic omnivore which can predate on aquatic animals and fishes (Chen 2006, Ma and Shi 2017), which means they can likely outcompete native turtles (Polo-Cavia et al. 2010). Third, exotic turtles may compete for microhabitats with native species. For example, *T.s.elegans* outcompetes native turtles for basking sites (Cadi and Joly 2003) and in experiments containing groups of native turtles and *T.s.elegans*, the native turtles experienced higher mortality (Cadi and Joly 2004).

**Current status and trends.** There have been no plans in Taiwan to remove this species from the wild, or to investigate its impacts. The importation and trade continues, with at least hundreds of thousands of young turtles being imported every year. In recent years, the government has invested heavily in development along the river, which has caused dramatic habitat loss on the riverbank. These constructions destroy nesting sites for native turtles located near the river banks. Since *T.s.elegans* tends to lay their eggs on muddy lands some distance from the riverside, there is likely higher survival rates of these nests leading to potential population replacement of the invasive species over native species (T-HC, pers. obs.).

Based on capture records (Chen 2006), the majority of this species now occurs in disturbed water bodies near urban areas, which indicates that their populations have not yet expanded to more natural environments. In most invaded regions, the population size has not yet exceeded *M.sinensis*, which is the most abundant native species (Chen 2006). The reason might be due to the low reproductive success and low survival rate for the hatchlings in the wild (but see Fig. 4C for the case of successful breeding in the wild), and secondly due to the fact that most pet keepers are not able to raise the imported young turtles to their adult size. Under this situation, removal of the adults might be effective in reducing their population size in the wild. A more a permanent solution would be to cease the trade of this turtle. Eliminating this species from the wild would be difficult but possible, largely depending on serious coordination and will of the government.

### *Physignathuscocincinus* (Cuvier, 1829)

**Figure 5.**
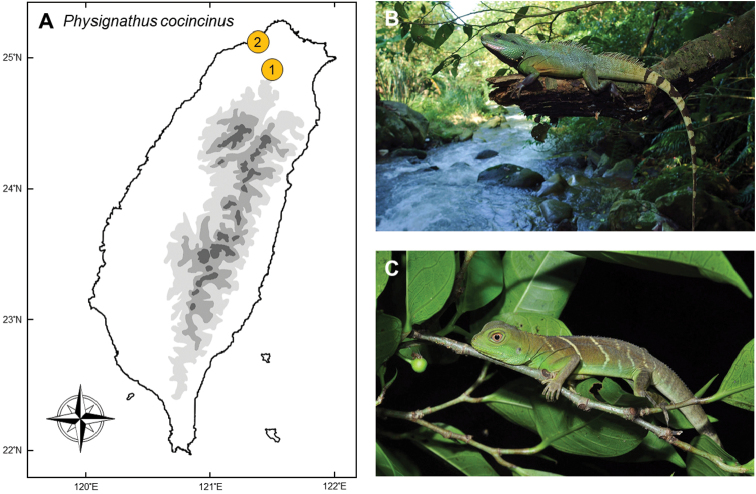
**A** The invasive population of *Physignathuscocincinus* was first established by an intentional release in Xindian (**1**), New Taipei City; and further transferred to Linkou (**2**), also believed to be intentional **B** the typical habitat of this semi-aquatic agamid is beside lowland streams **C** juveniles tend to rest on branches at night. Photographed by Ren-Jay Wang.

**Natural distribution.** This large-sized agamid lizard (Fig. 5) is widely distributed across southern border regions of China and Indochina, such as Myanmar, Laos, Thailand, Cambodia, and Vietnam (Vassilieva et al. 2016). They have invaded Hong Kong (To 2005), Penang of Malaysia (Grismer 2011b), and Florida, USA (Ferriter et al. 2009), all believed to be through animal trades. It has not yet been assessed under the IUCN Red List, but the Chinese government has listed this species under China Species Red List (Wang and Xie 2009) due to high intensity hunting for animal trades.

**Colonization history.** The first Taiwanese population of *Physignathuscocincinus* was discovered in Ankeng, New Taipei City in 2010 by a deliveryman who saw an adult lizard basking on the road along a river (point 1 in Fig. 5A). Soon after, some reptile keepers and students confirmed that *P.cocincinus* had established a breeding population in this area. Five years later (in 2015), some “giant green lizards” were reported around Linkou District of New Taipei City (point 2 in Fig. 5A), 20 km away from Ankeng and spaced by dense urban areas, which were later identified as a second population of *P.cocincinus*. This species is confirmed to have been breeding in these two streams to the present.

Since the core zone of both these invasive populations are in wild, torrential streams which are far from human settlements, they are thought to be established by intentional release. In the late 20^th^ century, *P.cocincinus* was valued as an alternative pet to the Green Iguana (*Iguanaiguana*) when the latter was prohibited by the Conservation Act of Taiwan. In 2001, captive breeding individuals of *I.iguana* began to be legally imported, which made *P.cocincinus* became practically worthless. The origin of the Ankeng and Linkou populations are suspected to be due to releases by pet traders.

**Threats to native species and ecosystems.***Physignathuscocincinus* is omnivorous, but primarily feeds on insects and snails (Manthey and Grossmann 1997, Ciou 2015). Research showed that adult *P.cocincinus* of the invasive population sometimes preys on native agamid lizards (*Diplodermaswinhonis* or *D.polygonata*), frogs (*Buergeriarobusta*), snakes (*Calamariapavimentata*), and mice (Ciou 2015) and thus could be a threat to many native animals along the stream systems, due to its large body size (SVL up to 250 mm and CL up to 650 mm (Vassilieva et al. 2016).

**Current status and trends.** The population in Ankeng did not initially show signs of quick spread because they were usually confined to riparian habitat along streams. During this period, some students, herpers, and pet keepers teamed up to remove this species from the wild. From 2013 to 2017, the government of New Taipei city further held projects to attempt to intensively remove this species. According to these surveys, more than 680 individuals were captured in Ankeng (Ciou 2015), with those removed by other citizens not included in this number. Removal of the Linkou population was conducted in 2016 and 2017, where approximately 200 individuals were removed. Research conducted at the time found that the population size could be effectively controlled with this intensity of removal, but would require consistent support from the government. Because of the species depends on streams, it might be possible to eradicate this species if the removal projects can be consistently sustained. We suggest that continuous support for these removal actions is a high priority with good chance of success, before the population spreads further and becomes impossible to remove. Actions to prevent further deliberate release or transportation of this species to other drainages should also be enacted.

### *Chamaeleocalyptratus* (Duméril & Bibron, 1851)

**Figure 6.**
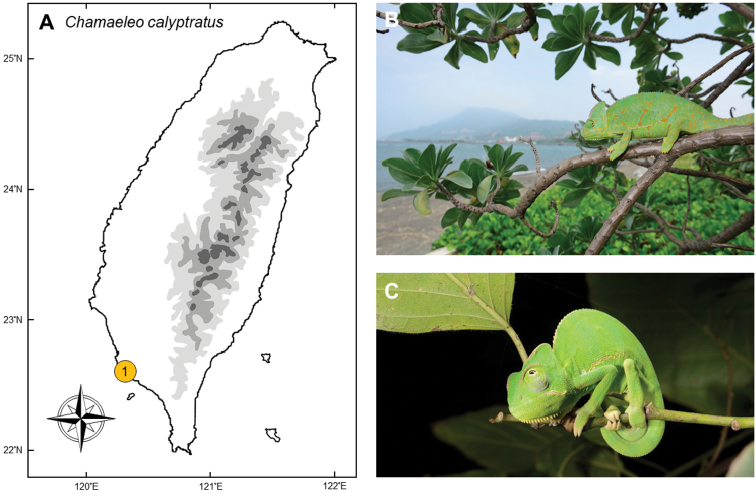
**A** The exotic population of *Chamaeleocalyptratus* was established by intentional release in Cijin (**1**), Kaohsiung City **B** a female exhibiting its mature coloration in their invasive site **C** neonates provide evidence of successful breeding in the wild. Photographed by Chung-Wei You.

**Natural distribution.** This large-sized Chamaeleon (Chamaeleonidae; Fig. 6) inhabits the tropical forests of south and southwestern Yamen, as well as southwestern Saudi Arabia (Tilbury 2010). This species has been introduced to Florida and Hawaii, USA (Krysko et al. 2004, Kraus and Fern 2004). In its native range, it faces a threat from collectors as it is a popular pet in wildlife trades. Although it is listed as “Least Concern” by the IUCN (LC; IUCN 2012), it is listed under CITES Appendix II as its trade should be closely controlled.

**Colonization history.** This species was first found on Cijin Island, ca. 200 meters offshore of Kaohsiung (point 1 in Fig. 6A), spotted along a beach by members of the public in 2011 and 2012 who uploaded photos of the animals on internet forums. At the time, these were considered as accidental escapes of pets, but another individual was captured during a fauna survey in Cijin in 2013 and further individuals have been found in the area since, including adults and adolescents (Fig. 6B, C).

Since the core zone of the chameleon population is located at the tip corner of an isolated peninsular, this invasive population was thought to be established by intentional release. As a popular and valuable animal in pet trades, captive breeding of this species is nevertheless difficult and costly. It was thus deduced that local pet traders released individuals deliberately so that they could “harvest” the young regularly and easily from the wild.

**Threats to native species and ecosystems.***Chamaeleocalyptratus* feeds mainly on insects, although large adults can prey upon small mammals and fledgling birds (Krysko et al. 2004). The invasive population of *C.calyptratus* is now restricted to Cijin Island, where invasive species of cockroaches are the most abundant prey item. Although there is no evidence of further spread, they might compete with native tree lizards (*Diploderma* spp.) for food and habitat if the species is to establish and spread in the future.

**Current status and trends.** This species is currently restricted to a hill located on the northwestern corner of Cijin. Although eggs have never been found in the wild, hatchlings and juveniles have been found to constitute a large proportion of the population. Many gravid females have been captured with fertile eggs. Thus it is considered that this species has established a breeding population on the island.

No official project has been stablished to remove this population. However, news of their appearance attracted numerous students, reptile keepers, and pet traders to the island to attempt to catch this valuable pet in the summer of 2013 and 2014. This resulted in the population size decreasing. This species is now difficult to find there, which suggests that hand removal might be an effective management option.

Because this area is connected to Kaohsiung city only by ferry and an underwater tunnel, the spread of this species is likely to remain limited within the island. Nevertheless, invasion risk persists elsewhere with deliberate release from the pet trade.

### *Iguanaiguana* (Linnaeus, 1758)

**Figure 7.**
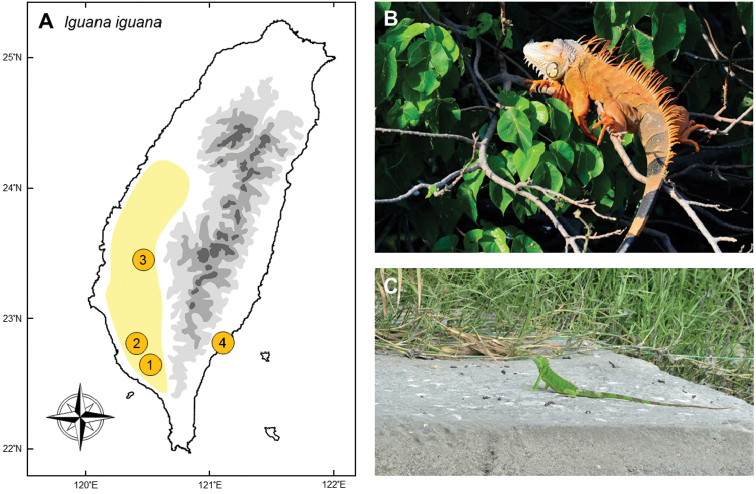
**A** The invasive populations of *Iguanaiguana* were originally established by multiple intentional release events, specifically in Pingtung (**1**), Kaohsiung (**2**), Chiayi (**3**), and gradually expanded to become a continuous distribution. In 2018, a small disjunct population occurred in Taitung (**4**), which might be another human-induced translocation event **B** a mature male occupying the canopy during courtship exhibition **C** the large number of young lizards demonstrates breeding success. Photographed by Chung-Wei You.

**Natural distribution.** This iguanid lizard (Fig. 7) is common in Central and South America, from south Mexico to Paraguay. It is a popular pet and has invaded many places, such as Florida (King and Krakauer 1966), Hawaii (McKeown 1996, Lever 2003), Fiji (Harlow and Thomas 2010, Thomas et al. 2011), Ishigaki Island of southern Ryukyus (Mito and Uesugi 2004; Falcón et al. 2013), and a large proportion of the West Indies (Falcón et al. 2012, López-Torres et al. 2012, Vuillaume et al. 2015). The invasive population in the West Indies has caused serious economic and ecological damage (Sementelli et al. 2008, Falcón et al. 2012). The species has not been assessed under the IUCN Red List but has been listed under CITES Appendix II, which limits the export of this species.

**Colonization history.** Although a popular pet in international reptile trade, keeping *Iguanaiguana* was illegal in Taiwan until 2001 when the first captive bred individuals were legally imported. During 2002 to 2007, tens of thousands of green iguanas were imported into Taiwan each year (CITES trade database). In 2004, some juvenile *I.iguana* were found in the wild and sent to Pingtung Rescue Center, suggesting that some individuals had escaped from the pet trade.

Establishment of invasive populations in Taiwan originated from several independent incidents (Fig. 7A). The populations in Wandan of Pingtung County (point 1 in Fig. 7A) and Niaosong Wetland of Kaohsiung City (point 2) were the first two, which are thought to have originated from intentional releases by local breeders. Individuals from Niaosong were then captured, sold, and released to Bazhang River of Jiayi County (pint 3), and Rende of Tainan City. Recently, newly established populations were found in Yunlin, Changhua, and Taichung Counties. A road killed individual and some living ones were caught in Taitung in 2018 (point 4), but whether it has established a population there remains unknown. Similar to the case of *Chamaeleocalyptratus*, local pet traders are suspected to have released this species intentionally for future harvesting.

**Threats to native species and ecosystems.** According to the experience of the Great Caribbean Basin, *I.iguana* can reach huge population sizes in suitable habitats (Falcón et al. 2012). Normally, they prefer forest edges near streams or rivers (Meshaka et al. 2004). Therefore, the tropical monsoon forests in southwestern Taiwan provides suitable habitat, and they are considered likely to expand to large numbers.

Based on analyses of stomach contents, invasive *I.iguana* populations in Taiwan feed mostly on *Broussonetiapapyrifera* (Rosales, Moraceae), one of the most abundant shrubs in the disturbed areas of Taiwan. Although we do not have evidence on the threats to native ecosystems in the wild, human agriculture might be seriously damaged from adult iguanas which are able to wipe out the entire crops from the field within a few days. Digging burrows along river banks creates damage to the structure of irrigation channels, which can make structures unstable and threaten the safety of nearby citizens (Sementelli et al. 2008; Falcón et al. 2012). Female iguanas commonly use graveyards which causes damage to tombs, then interpreted as bad omens by the local people (interview records from local people).

**Current status and trends.** This species first established disjunct populations in southern Taiwan, and then gradually invaded into central Taiwan. During the invasion process, subordinate males play the role of dispersers into novel habitats at the invasion fronts, where they then occupy a territory and become dominant males (Fig. 7B). Females are then attracted by these males to the newly invaded sites (T-HC, pers. obs.). Compared to *P.cocincinus*, this species inhabits higher canopy in woodlands, which makes them difficult to be caught or to be removed. Accidental or deliberate releases by pet trader further make their spread out of control. Continuous captures of young individuals over time (Fig. 7C) suggests the feral populations of this species are consistently growing.

The Chiayi City Government has offered rewards for invasive *Anolissagrei* for several years and *I.iguana* was included in this rewards program in 2017. However, this approach is considered ineffective by scientists as it has not resulted in population decreases of either of these two species. In southern Taiwan, Kaohsiung City Government conducted another project to evaluate the invasion of *I.iguana*. More than 2,200 adults were caught in Kaohsiung and Pingtung counties from 2013 to 2017 by T-HC’s laboratory members, and this seems to have effectively reduced the population size (T-HC, unpublished data). We suggest that removal should focus on mature individuals near nesting sites before the breeding season, because dominant adults display strong habitat loyalty during this period (T-HC, pers. obs.). A large proportion of the captured individuals from the government reward program, however, were young lizards which naturally have very low survival rate in winter (T-HC, pers. obs.), which made this program inefficient. We conclude that complete eradication is unlikely in Taiwan; but more efficient management policy could help to depress their population.

### *Anolissagrei* Duméril & Bibron, 1837

**Figure 8.**
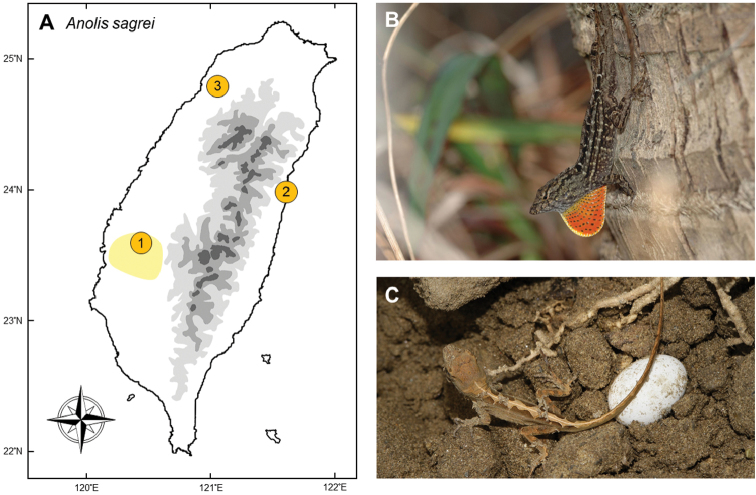
**A** The population of *Anolissagrei* was first discovered in Jiayi (**1**), southwestern Taiwan. Subsequently, this lizard occurred long-distance dispersal to eastern (Hualien (**2**)) and northwestern (Hsinchu (**3**)) Taiwan **B** a mature male showing courtship exhibition on a trunk in the invasive region **C** an egg and a hatchling of *A.sagrei*. Photographed by Ren-Jay Wang (**B**) and Wen-Bin Gong (**C**).

**Natural distribution.** This small-sized anole (Dactyloidae; Fig. 8) is widespread across the islands of Bahamas, Cuba (Campbell 1996), Honduras (Rodríguez Schettino 1999), and several islands nearby, such as Swan Island (Rodríguez Schettino 1999), Cayman Brac and Little Cayman (Losos et al. 1993). Although it seems that this species is widely distributed in Central America and the Caribbeans, many of the populations, including those in Jamaica, Grand Cayman (Roughgarden 1995), Belize (Rodriguez Schettino 1999), Grenada (Greene et al. 2002), and the East Coast of Mexico (Conant and Collins 1998) are invasive. It is also introduced to several regions of the USA, for example, Florida, Texas (Conant and Collins 1998), Louisiana (Steven and Lance 1994), Georgia (Campbell 1996), and even Hawaii (Kishinami and Kishinami 1996). Recently, it has been reported to have invaded Singapore, possibly with imported plants (Tan and Lim 2012). This species is not listed under any endangered species legislation and frequently appears on alien species lists in many countries.

**Colonization history.** The first record of this species was in September of 2000, when one female and two males were found beside a road near a plant nursery in Sanjiepu, Chiayi by Gerrut Norval (point 1 in Fig. 8A). These individuals were captured and sent to the Senckenberg Museum for confirmation of identification. In November 2000, 28 individuals were collected in the same area, and preserved in the National Museum of Natural Science (Norval et al. 2002).

It remained unknown how this species entered Taiwan, but we deduce that potting compost imported to the nursery likely contained eggs of this species, as was observed during its invasion onto Guana Island (Perry et al. 2006). The most likely source of this population is thought to originate from Florida, because of the similarity in parasite composition and mitochondrial sequences (Kolbe et al. 2004, Norval et al. 2011). In 2006, this species was first recorded in Hualien City (Chang 2007), with a huge population in Chicingtan and several satellite populations such as Hualien City and the campus of Dong Hua University. Around 2014 – 2015, a third population was discovered in Hsinchu. This species is now confirmed to occur in three localities: Chiayi, Hualien, and Hsinchu (Fig. 8A).

*A.sagrei* expands quickly once introduced to new areas and may adapt to new environments well due to its high genetic variation (Losos et al. 1993, Gerber and Echternacht 2000, Kolbe et al. 2004). An investigation in 2007 showed that *A.sagrei* occurred in more than a quarter of the sampling sites in Santzepu, Chiayi (Hou et al. 2007). After six years, the distribution of this species became even wider, and one third of the sampling sites in Santzepu was reported to be inhabited by this species (Wang 2013).

**Threats to native species and ecosystems.***Anolissagrei* occupies the tree-trunk niche within its habitat (Fig. 8B). In invaded zones, this species reaches tremendous numbers with extremely high population density, and is probably capable of out-competing other arboreal insectivorous vertebrates. In Hualien, populations of the grass lizard *Takydromusluyeanus* (Lacertidae) have declined dramatically in sympatric sites (Yang 2017), while their impact on the tree lizard *Diplodermaswinhonis* (Agamidae) remains unknown and needs further monitoring. Predation by the species has also altered communities of invertebrates, particularly ants (Huang et al. 2008).

**Current status and trends.** In order to persuade citizens to help remove the lizards, the Chiayi County Government has offered rewards for carcasses of the anoles since 2009. However, this policy was regarded as being inefficient. The rewards have encouraged locals to accumulate huge amounts of carcasses, but this has not been effective in removing the population. We suggest several reasons for this: first, most citizens try to catch the lizards from the core zone(s) of the invasion, where high densities of lizards facilitate people to earn the reward with the least effort. However, individuals can quickly fill these gaps from adjacent regions and the population is thus impossible to eliminate. Second, with a long breeding season and continuous clutch production, it is ineffective when only a low proportion of individuals are removed. Although huge amounts of money have been spent on removing individuals every year, the distribution of this species is still expanding rapidly in western Taiwan. In contrast, the research team in Hualien, eastern Taiwan used an alternative strategy. Instead of citizens, volunteers were trained to focus on invasion fronts. By removing individuals from the front, the team led by Dr. Yi-Ju Yang has successfully reduced the speed of the invasion, and successfully eliminated some newly established populations. To date, the Chiayi population is continually expanding, but the expansion in Hualien has been slowed.

Current evaluations indicate that the expansion of *Anolissagrei* is unstoppable and that regions which have already been invaded, eradication is likely impossible. The only thing we can do is to slow down the expanding speed of the front. Transportations of potted plants from core regions of lizards should be quarantined (Campbell 1996, Norval et al. 2017). Maintenance or restoration of the natural habitats can also help with stopping the spread of this species in rural areas.

### *Gekkogecko* (Linnaeus, 1758)

**Figure 9.**
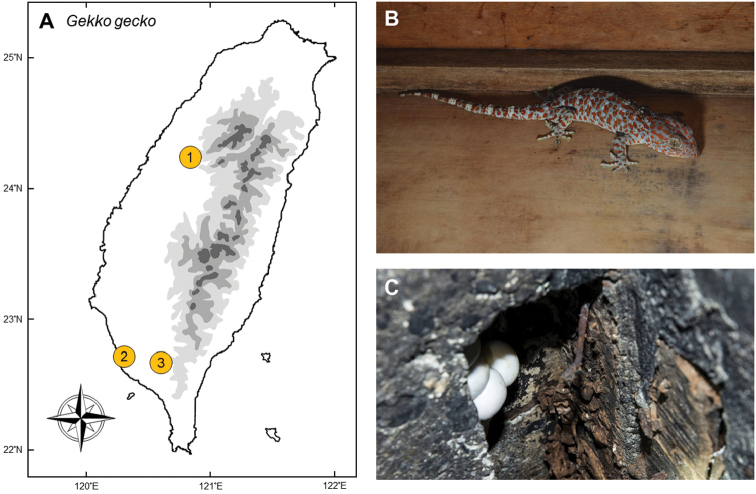
**A***Gekkogecko* has been discovered in several disjunct localities (Taichung (**1**), Kaohsiung (**2**), Pingtung (**3**)), which was thought to be from multiple release events **B** a mature gecko showing defensive posture on a cornice in Kaohsiung **C** eggs in a nearby cave. Photographed by Ren-Jay Wang (**B**) and Ko-Huan Lee (**C**).

**Natural distribution.** This large-sized gecko (Gekkonidae; Fig. 9) is widely distributed in southern Asia, including southern China, Indochina, Malaysia, Indonesia, and the Philippines (Rösler et al. 2011). It has been reported to have invaded the Caribbean (Henderson et al. 1993), Hawaii, Florida (Kraus 2009), Belize (Caillabet 2013), Madagascar (Das 2015), and Brazil (Júnior 2015). This species has not yet been assessed under the IUCN Red List, and it is not listed under CITES.

**Colonization history.** Early records of this species in Taiwan can be traced back to the Japanese colonial period (Okada 1936). Two records of this species have been documented, both with only a single individual; one occurred in the botanic garden in Taipei, the other was caught by a junior high school student in Tainan. Because of its rareness, this species was listed in the first version of the Conservation Act in the 1980s. However, herpetologists now regard these records as accidentally imported individuals and removed this species from the protected list.

Rediscovery of this species occurred in 2008, when five individuals were found in Taichung (point 1 in Fig. 9A) (Norval et al. 2011). In 2013, an egg litter and a hatchling of this species were discovered by Ming-Hung Hsu and Chi-Yu Huang in a fauna survey in Kaohsiung, representing the first breeding population ever found in Taiwan (point 2). In addition to Kaohsiung, it has also been sporadically reported in several other places such as Neipu township of Pingtung (point 3). This species might have been carried to these areas accidentally either by cargo ship or escaped from pet owners.

**Threats to native species and ecosystems.** We have recorded individuals regurgitating invasive species of cockroaches after being captured. Therefore, we suspect that they prey mainly upon cockroaches around houses, with some other small invertebrates and vertebrates. Besides direct predation, *G.gecko* may compete with other native geckos.

**Current status and trends.** Although distributed sporadically in a few places, only the population in Kaohsiung has been confirmed as a reproducing population. Distribution of this population is restricted to Guishan hill near Lienchi Lake, Zuoying District. Individuals occur around buildings and nearby forests, which is similar habitat to that which this species uses in native areas. There is currently no specific program to eradicate the species. However, the population size has been depressed through spontaneous capturing programs organized by students and pet keepers. Fortunately, Guishan is isolated from nearby natural habitats by urban areas which might prevent *G.gecko* from spreading to other natural habitats. However, a comprehensive survey is still required to investigate the dynamics of this population, especially with the risk that pet keepers might release more individuals to other localities.

### *Gekkomonarchus* (Schlegel, 1836)

**Figure 10.**
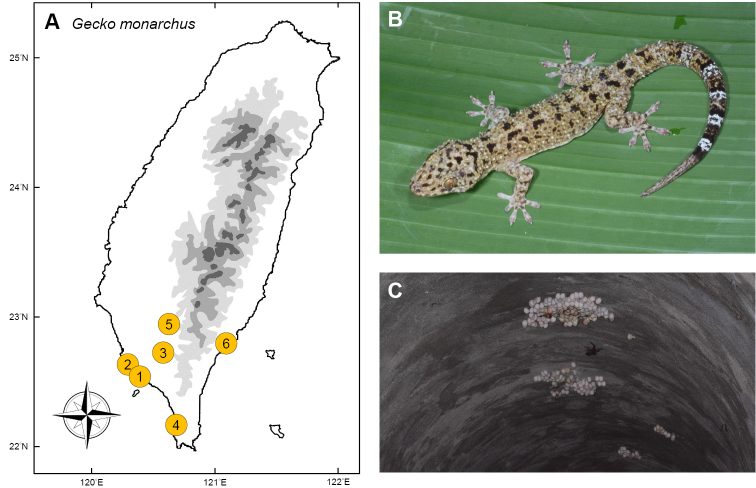
**A** Invasion of *Gekkomonarchus* is thought to have occurred from international timber trades near the Kaohsiung Harbor ((**1**) and (**2**)) and a log-processing area (**3**). In 2018, the newest population was found with disjunt distribution in Taitung County (**4**) **B** a mature individual **C** a large colony of eggs. Photographed by Gaus Shang.

**Natural distribution.** This medium-sized gecko (Gekkonidae; Fig. 10) occurs throughout the Philippines, Singapore, Peninsular Malaysia, reaching to southern Thailand (from Narathiwat to Surat Thani), Indonesia, including several islands (e.g., Aru Islands, Kei Islands, Ambon Island), and can be as far east as New Guinea (Pauwels and Sumontha 2007, Rösler et al. 2011). It has been reported to have been accidently imported to New Zealand and South Africa (Gill et al. 2001, Bauer and Branch 2004). This species has a wide distribution, and is locally abundant throughout most areas. It is not listed as threatened under any legislative acts.

**Colonization history.** This species was first discovered in 2009 from Linyuan District, Kaohsiung by locals (point 1 in Fig. 10A) and later identified and confirmed by Dr. Szu-Lung Chen (Shang 2013). In 2010, researchers of Observer Ecological Consultant Company recorded a second population in Fengbito, Kaohsiung (point 2). The third population in Neipu, Pingtung County (point 3) was reported by Ching-Gou Ji in the same year. In 2016, Shang et al. (2016) estimated that there were approximately 200 individuals in Linyuan, 1,000 in Fengbito, and 100 in Neipu. In 2017, this gecko further colonized Checheng (point 4) and Liugui (point 5). Since 2018, a newly established population was found in Taitung City (point 6), with a disjunct distribution far away from the western populations. In addition to these populations, sporadically caught individuals were also reported from Renwu in 2012, Chiayi in 2012, Yilan in 2012, and Nantou (2013).

How this species entered into Taiwan remains unknown, but it is thought to be related to the timber trade of Kaohsiung Harbor (point 1 and 2 in Fig. 10A), as two of the three originally invaded localities are near industrial zones. The species lives close to humans in its native range (Grismer 2011a, b), and is thus likely to gain access to importation cargos from southeast Asia. New localities have been reported far from the originally colonized areas every year, which implies that this species is likely invading these areas through human mediated dispersals.

**Threat to native species and ecosystems.***Gekkomonarchus* eats small invertebrates in its native range. In Taiwan, it preys primarily upon Coleoptera and Blattodea (Shang et al. 2016), with small snails, egg shells and seeds also occasionally recorded from stomach contents. In invaded regions, this gecko out-competes other geckos such as *Lepidodactyluslugubris* on Pulau Cebeh (Grismer 2011a). This species can occur in high densities in a variety of habitats, and can be the most dominant lizard on some islets (e.g., Cebeh of Seribuat Archipelago) (Grismer 2011a). As a result, this species is capable of dominating many lowland habitats and possibly wipe out native geckos.

The most crucial task in the near future would be preventing this species from moving onto Orchid Island, an offshore islet with only 48 km^2^, which is occupied by *Gekkokikuchii* (Oshima, 1912), a species closely related to *G.monarchus* and confined to this island within Taiwan (Siler et al. 2014). *Gekkokikuchii* is similar with *G.monarchus* in body size, predicting large niche overlap with each other, once they occur sympatrically. Thus, if invasion of *G.monarchus* occurred on Orchid Island, it is thought probable to wipe out the population of *G.kikuchii*.

**Current status and trends.** In Taiwan, this species lives close to humans and disturbed areas such as buildings or tunnels (Shang et al. 2016). Shang et al. (2016) estimated the population size of Fengbito to be 5,029 individuals using mark-recapture methods. A large proportion of individuals inhabit military tunnels beneath subtropical forest, which makes them difficult to be eradicated.

An eradication program was conducted by the Forest Bureau from June to December, 2015. A total of 532 individuals were caught, with more than 4,000 eggs being destroyed from three main invaded regions, mostly from Linyuan (Shang et al. 2016). Shang et al. (2016) suggested that removal plans should continue to restrict the population size, and to stop the invasion progress. However, the government seems unwilling to continue the program to eradicate this species. Based on current situation, it has a high potential to spread widely through southern Taiwan within a short period.

### *Hemidactylusbrookii* Gray, 1845

**Figure 11.**
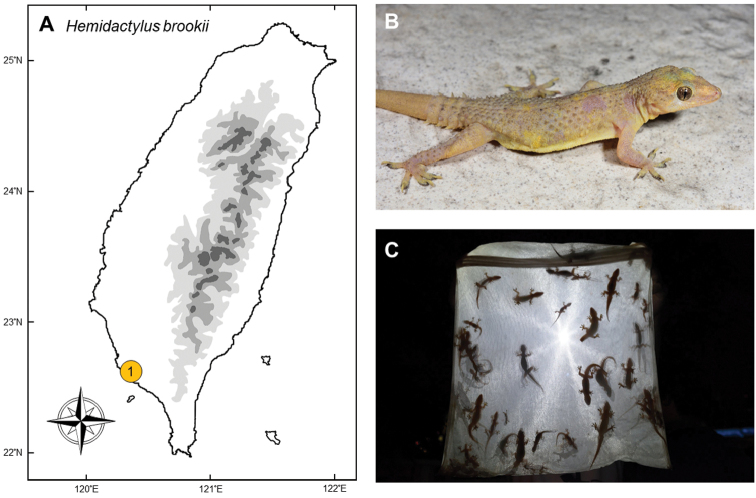
**A***Hemidactylusbrookii* is the most-recent invasive species which was discovered from a single population along the river banks of Love River, Kaohsiung City, southern Taiwan (**1**) **B** a mature male **C** large amount of young geckos indicated that they have successfully colonized in the city. Photographed by Chung-Wei You.

**Natural distribution.** This small-sized gecko (Gekkonidae; Fig. 11) is widely distributed in Central America, tropical Africa, Asia, including the Indian subcontinent, Indochina and Indonesia (Kluge 1969, Bauer et al. 2002). Owing to the extremely wide-range distribution, the taxonomy, phylogeography, and invasion status of *Hemidactylusbrookii* sensu lato was controversial and attracted the interests of researchers (Bauer et al. 2010, Mahony 2011, Lajmi et al. 2016). Currently, the nearest population is in Hong Kong, which was described as an invasive species (Romer 1977). It is not listed as threatened under any legislative acts.

**Colonization history.** This recently discovered species was found along the river banks of the Love River in Kaohsiung City (point 1 in Fig. 11A). June 2018, a college student Dong-Long Yeh took some photos from an unidentified gecko. He sent these photos to Chung-Wei You, a herpetologist who has plenty of experiences to observe herpetofauna worldwide. Although it was an individual with a regenerated tail, it was identified as a Brooke’s house gecko (*Hemidactylusbrookii*) by You. He organized a team in July 2018 to investigate whether it had established a population along the river side. During this survey, they identified and spotted numerous Brooke’s house geckos along the river, and captured 36 individuals including eight males, eleven females, and 17 juveniles. Large numbers of juveniles indicated that this gecko has successfully colonized in the city (You 2019). It remains unknown how and when this species invaded Kaohsiung. It was suspected that this species was introduced by cargo ships from nearby harbors.

**Threat to native species and ecosystems.** Feces of *H.brookii* were collected to identify its diet in Kaohsiung. Diverse insects were identified using microscope, including Coleoptera, Orthoptera, Hemiptera, Diptera, Dermaptera, and Araneae, on which endemic geckos also prey (You 2019). The influence of *H.brookii* on local ecosystems and whether it competes with native or other invasive geckos remains unstudied.

**Current status and trends.** This species mainly dwells in the cement river bank along the Love River, and occasionally spotted in the bushes. A large population was found sympatric with native *Gekkohokouensis* and suspected invasive *Hemidactylusfrenatus*. The large number of juveniles seen in this population suggests that this species has been breeding in this area, despite no eggs and gravid females were found during the survey. Based on this observation, You (2019) further deduced that *H.brookii* has a high potential to spread along the river bank to nearby urban or suburban regions. Since this is a newly discovered species, there is no eradication program to control the population size.

### *Eutropismultifasciata* (Kunl, 1820)

**Figure 12.**
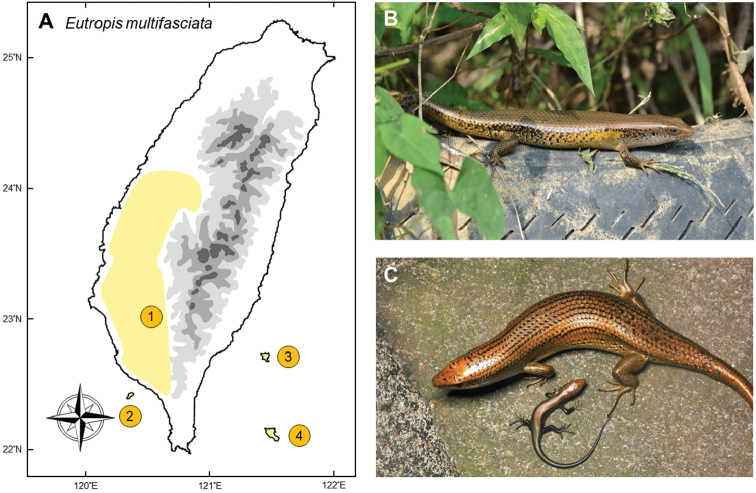
**A***Eutropismultifasciata* has originated from Meinong (**1**), expanded to the entire southwestern Taiwan, and also colonized offshore islets such as Siao Liouciou (**2**), Green Island (**3**), and Orchid Island (**4**) **B** an adult male basking on an abandoned tire along a river bank **C** a mature female with her new-born baby. Photographed by Chung-Wei You (**B**) and Ren-Jay Wang (**C**).

**Natural distribution.** This medium-sized skink (Scincidae; Fig. 12) has a wide distribution in eastern Asia, from India to southern China, including Thailand, Myanmar, Laos, Cambodia, Vietnam, and Malay Peninsula. It is also common on some islands of the Philippines, Indonesia, and New Guinea (Vassilieva et al. 2016). It has also invaded Florida, USA (Meshaka 1999). It is not listed as threatened under any legislative act.

**Colonization history.** This species was first recorded in Meinong District and Chengcing Lake, Kaohsiung in 1992 (Ota et al. 1994) (point 1 in Fig. 12A) by Prof. Hsueh-Wen Chang and his lab members and thought to have had already established a breeding population prior to this time. It was thought likely to be introduced into Taiwan by cargo ship, presumably through the international timber trade. Two years after its first discovery, populations were found in Fongshan, Ciaotou, and Chaujou (Chang and Liu 1995). During the period of 1999 to 2002, fauna surveys conducted by Endemic Species Research Institute showed that this species had expanded its population southward to Fangliau, northward to Rende (Lin 2008, Tseng and Lin 2008), and also successfully colonized Siao Liouciou (point 2 in Fig. 12A), a tiny islet located 15 km from the western coastline of Taiwan (Shang 2001). By 2007, the invasion front had extended from Cigu to Dounan, although it seemed to stop on the southern side of the Jhuoshuei River (Tseng and Lin 2008), a new, isolated population of *E.multifasciata* was found in a submontane area of Puli (the northern side of Jhoushuei River) in 2014.

Although all of these localities are in western Taiwan, Green Island (point 2) and Orchid Island (point 3), located 33 and 72 km off shore from the east coast of Taiwan, have been reported to contain populations of *E.multifasciata*. The first record of this species on Green Island was a carcass, presumably killed by cats, in 2008. In the same year, Researcher Te-En Lin, confirmed that a population consisted of approximately one thousand individuals had successfully colonized around the Green Island lighthouse. On the other hand, *E.multifasciata* has been recorded for several years on Orchid Island with the population size not well documented and time of invasion unknown.

Whether this species immigrated to Green Island and Orchid Island through natural dispersal or artificial introduction remains controversial. For instance, previous research on reptiles (Ota and Huang 2000, Siler et al. 2014), birds (Oliveros and Moyle 2010) and beetles (Tseng et al. 2018) indicated a northward stepping-stone dispersal model across the Taiwan-Luzon volcanic belt. Furthermore, Kurita and Hikida (2014) revealed that the Ryukyu Five-Lined Skink could disperse northward via the Kuroshio Current. Further research is needed to verify the source and mode of introduction of *E.multifasciata* to Green and Orchid Islands.

**Threat to native species and ecosystems.***Eutropismultifasciata* is a viviparous skink which breeds all year round with 4–12 neonates per litter (Chang and Liu 1995, Shang et al. 2009). Such productivity gives this species an advantage to adapt to disturbed regions and further expand its population. *E.multifasciata* occupies a variety of habitat types in southwestern Taiwan, including coastal areas, open forests, rural grasslands, disturbed lowland areas, and submontane areas, but prefers living in ditches and water channels. The ability to dive into water when encountered by predators also serves to provide them a better chance to explore novel habitats. By being semiaquatic, this species can spread quickly by using irrigation throughout agricultural lands.

Scientists suspect that the congener *Eutropislongicaudata* would be the first native species to be impacted from the invasion, because *E.multifasciata* has a much higher fecundity than *E.longicaudata*. *E.longicaudata* laid an average of ten eggs three times annually, while *E.multifasciata* can give birth to 4–12 hatchlings up to five times every year (Chu 2000). To date, it appears that habitats previously occupied by *E.longicaudata* have gradually been replaced by *E.multifasciata* (K-HL, pers. obs.). Their invasion is also likely having serious impacts on several endemic skinks, such as *Plestiodonleucostictus*, which was recently elevated to species status from subspecies (Kurita et al. 2017) and has a unique color morph on Green Island (Hikida 1988), suggesting a distinct evolutionary unit for this population.

**Current status and trends.** In the early 20^th^ century, *E.multifasciata* had been one of the major targets of government-funded monitoring. It now appears to be impossible to eradicate, with *E.multifasciata* having become one of the most abundant skinks south of the Jhuoshuei River, with the highest population density being in southern Taiwan (Tseng and Lin 2008). Endemic Species Research Institute has attempted an eradication program on Green Island where its habitat significantly overlaps with *P.leucostictus*, but the population still persists. The northward expansion of this species is still ongoing and thought to be largely unstoppable.

### Species with a controversial status

#### *Lithobatescatesbeianus* (Shaw, 1802)

**Figure 13.**
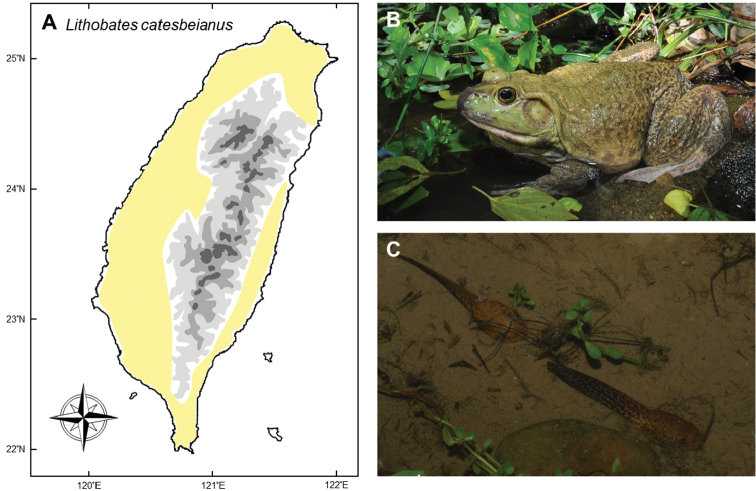
**A** Escaped or released *Lithobatescatesbeianus* has been recorded almost all around Taiwan **B** the injured snout of this adult indicated it is recently released from captivity. However, it seems that they have not established a successful breeding population **C** one of the very rare cases of tadpoles found in the wild was discovered by Gaus Shang. Photographs by Ren-Jay Wang (**B**) and Gaus Shang (**C**).

**Notes.** Captive breeding of this large ranid frog (Fig. 13) for food started in the 1950s in Taiwan. Nowadays, tens of thousands of bullfrogs are sold from commercial farms every year. Occurrence of the bullfrog in the wild is a common consequence of intentional release for religious mercy ceremonies. Although likely to have occurred much earlier, this situation was not noticed until the late 1980s. Since first included in the amphibian guide by Lue and Lai (1990), most guide books and fauna reports have included the bullfrog as an invasive species in Taiwan (Shang et al. 2009, IUCN SSC Amphibian Specialist Group 2015).

Mature individuals, froglets, and tadpoles are all potential targets for release ceremonies. Therefore, a variety of frog sizes have been discovered in the wild. Nevertheless, despite common records around the low land habitats of Taiwan (Fig. 13A), successful breeding by the frog in nature is rare, possibly due to the higher temperatures in Taiwan compared to its native range (see a comparison between native and invasive regions; Degenhardt et al. 1996). Some released adults (Fig. 13B) survive for a while and produce breeding calls, but most of them do not breed. Most froglets die soon after release, and there is no evidence to show that these frogs can reach sexual maturity in the wild, although the reasons why are poorly understood. In 2009, Gaus Shang recorded a group of tadpoles near Xindian Stream, New Taipei City (Fig. 13C), representing one of the very few cases of suspected breeding by this frog in the wild, but the population did not persist. This suggests that the bull frog is capable of breeding in Taiwan, although a breeding population has not yet been recorded to have successfully established. Because of the limited evidence for their reproduction in the wild, the status of this species should perhaps be revised to reflect that they are continuously released into the wild, rather than constituting an actual “invasive” population. While the failure of breeding in the wild is suspected to be a consequence of the relatively higher temperatures in Taiwan (Degenhardt et al. 1996), the mid-elevation areas in Taiwan have the potential to provide lower temperatures to support their breeding. Large water bodies at these regions should be monitored to prevent this species from establishing “real” breeding populations.

#### *Mauremysreevesii* (Gray, 1831)

**Figure 14.**
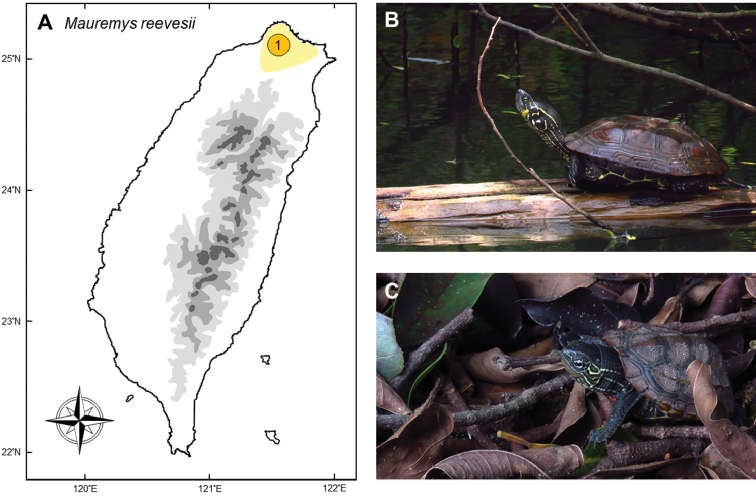
**A** Most confirmed records of *Mauremysreevesii* in the 20^th^ century are from the Tamsui River Drainage (**1**) close to the highly developed Taipei City, where this population has gradually gone extinction in the late 1980s **B, C** the pictures of the adult and the young were taken from a native population on Kinmen, an islet 3 km offshore from China. Photographed by Wei-Lun Lin.

**Notes.** This moderate-sized fresh water geoemydid turtle (Fig. 14) is distributed throughout central and eastern China and the Korean peninsula. Although the wild populations have experienced dramatic population decline due to commercial over-exploitation in China, the captive populations might have become one of the most common species in Chinese turtle farms. It has been introduced into Indonesia, Palau, Timor-Leste, Japan, and Ryukyu Archipelago (Lovich et al. 2011). The introduced population in Japan (Hikida and Suzuki 2010) has caused hybridization with the endemic *M.japonica* (Suzuki et al. 2013), and was thus regarded as a threat to the genetic integrity of the latter (Suzuki et al. 2011, Suzuki et al. 2013).

The first record of this turtle in Taiwan was reported by Horikawa (1934), who discovered this species in 1931 near Taipei Basin. Thereafter, most of the records occurred in Tamsui River drainage (Mao 1971) of Taipei, one of the most seriously polluted and disturbed rivers in Taiwan (Fig. 14A). The population size had never been evaluated until the late 1980s, when the first version of the Conservation Act of Taiwan listed it as a threatened species (Class III). The population in the Tamsui River seemed to have declined dramatically in the 1980s and 1990s. In the late 1990s and early 2000s, several thorough investigations (Chen and Lue 2010) indicated that this species might have gone near-extinction in Taiwan. In 2008, a revised list of protected species under the Conservation Act reevaluated this turtle as a “critically endangered species” (Class I), which was the highest rank among all reptiles in Taiwan.

Although currently listed as a threatened native species, this status has recently been challenged by Fong and Chen (2010) for a number of reasons. First, the discovery of this species in the 1930s was 70 years after the first systematic investigation of Taiwanese fauna when Robert Swinhoe visited the island in the 1860s. It is worth to note that the majority of herpetofauna has been uncovered before the early 1930s; until a new wave of new species and new records in the late 20^th^ century. The age of discovery of *M.reevesii* was not only much later than any other testudine, but also one of the latest among all herpetofauna in Taiwan before World War II. Second, confirmed individuals were restricted to the Tamsui Drainage (Mao 1971), a highly disturbed area close to the most developed city in Taiwan, Taipei City. Third, it is hard to explain why this species has never been found elsewhere in other natural drainages which retain much better environments. Together, these facts led Fong and Chen (2010) to suspect that this turtle is actually introduced by Chinese immigrants in the early 20^th^ century.

In order to trace the origin of *M.reevesii* of which the status was also controversial in Japan, Suzuki et al. (2011) used molecular approach to study the population genetics of this species; the samples comprised a native individual from Taiwan. Their results indicated that the genetic divergence among China, Japan and Taiwan populations was far below than the expectation deduced from other terrestrial taxa. Suzuki et al. (2011) thus made a conclusion which was congruent to Fong and Chen (2010), that both Japanese and Taiwanese populations of *M.reevesii* were originated from human release. This deduction was also referred in the review by Lovich et al. (2011).

The reason for the disappearance of this turtle in the Taipei Basin remains a mystery. Habitat destruction could be a major reason, while hybridization and backcross to the dominant native congener *M.sinensis* could be another, as mitochondrial sequencing has shown hybridization between the species and intermediate forms exist (Fong and Chen 2010, Chen and Lue 2010). Nowadays, *M.reevesii* individuals are occasionally found in other drainages of Taiwan, but have never proven to constitute a breeding population. These new individuals presumably originated from Chinese turtle farms and are likely to be released from pet keepers.

#### *Hemidactylusfrenatus* Dumeril & Bibron, 1836

**Figure 15.**
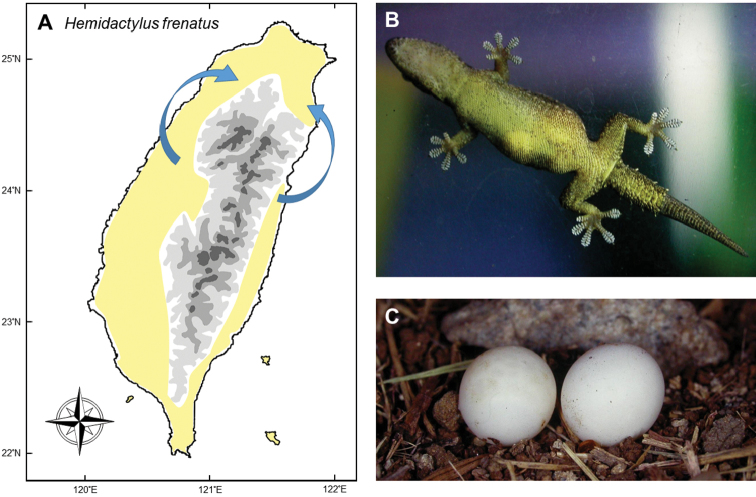
**A***Hemidactylusfrenatus* has expanded not only throughout the lowland of Taiwan, but also almost all islands in the west Pacific region. The northern one third of Taiwan is believed to have become occupied only in recent decades (indicated by arrows) **B** a gravid female **C** the eggs. Photographed by Si-Min Lin.

**Notes.** This small-sized, house-dwelling gecko (Fig. 15) is one of the most notorious, successful, and weedy invasive reptile in the global scale (Carranza and Arnold 2006, Behm et al. 2019). In Taiwan, it is not only the most common gecko, but perhaps the most abundant reptile on the island. Since first reported by Boulenger in 1885, it was always treated as a native species throughout the 20^th^ century. However, several lines of evidence, such as geographic genetic pattern (Moritz et al. 1993), or the range expansion in nearby regions (Hunsaker 1966, Case et al. 1994), suggest that most *H.frenatus* populations in the world are exotic. Another line of evidence came from the phylogeny of *Hemidactylus* spp. (Bauer et al. 2010), which indicated that *H.frenatus* belongs to a species-rich clade with all its members distributed exclusively in southern Asia. Ota et al. (2004) formally recognized the populations in Ryukyu Archipelago as invasive, and further interpreted that the current populations on most Oceanian and East Asian islands were similarly derived from human-associated dispersals. This was also emphasized in Kraus (2009), and this species is now considered invasive in many countries. Currently, the IUCN lists the occurrence of *H.frenatus* in Taiwan as an introduced population; the Global Invasive Species Database (GIBD) website categorizes it as “cryptogenic”, and treats adjacent regions (Japan and the Philippines) as introduced.

Historical observation indicated that *H.frenatus* and *H.bowringii* occupied the southern and northern parts of Taiwan, respectively (Ota 1989, Lin and Cheng 1990). However, *H.frenatus* has now expanded all around the island, including the northern third of the island which was not occupied by this species several decades ago (Ota 2009) (Fig. 15A). We have no evidence for their impacts on native species, but it is considered likely to compete with native *Gekko* spp., of which the genetic and species diversity is still under-estimated. Currently, most local people (including most biologists) do not yet realize its status as an introduced species; but regardless, eradication of this species from the wild seems impossible.

#### *Lepidodactyluslugubris* (Dumeril & Bibron, 1836)

##### *Hemiphyllodactylustypus* Bleeker, 1860

###### *Indotyphlopsbraminus* (Daudin, 1803)

**Notes.** These three small squamates share a common feature: parthenogenesis. They are all regarded as native species in the current literature, and we do not yet have sufficient evidence either to justify, or reject this status. However, the possibility that they are in fact invasive should be reconsidered based on accumulating new lines of evidences.

*Lepidodactyluslugubris* was first listed as a member of the fauna of Taiwan by Chen’s (1984) revised book, but under the name *Gehyravariegateogasawarasimae* Okada 1930, a junior synonym of *Lepidodactyluslugubris* which was used to refer to the population of Ogasawara Islands. However, Chen and Yu (1984) did not provide sufficient information for identification or the collection information of this species. Later in 1984, Ota (1986) collected and identified this gecko. Together with *H.frenatus*, *L.lugubris* is regarded as one of the two most globally successful “weedy” geckos (Carranza and Arnold 2006; Behm et al. 2019), invading a wide range especially in insular regions, such as Caribbean islands (Henderson et al. 1976, Kraus 2009, Lorvelec et al. 2011, Lorvelec et al. 2017). Colonization of this species in west Pacific islands was thought to be relevant to the active army traffic since World War II (Hunsacker and Breese 1967, Ineich 1999). The unstoppable expansion is still occurring to new places (Krysko and MacKenzie-Krysko 2016, Lapwong and Juthong 2018, Behm et al. 2019).

Similar to *L.lugubris*, *Hemiphyllodactylustypus* was discovered in central 1980s by Lue et al. (1985). However, it is hard to identify the status of these two geckos prior to this, because the investigation of lizards of Taiwan was scarce until the late 1970s. Not long after these records, Japanese scientists reported the occurrence of both two geckos in southern Ryukyu (200 km east of Taiwan) in the 1990s, and formally treated them as invasive species since the turn of the 21^st^ century (Ota et al. 2004). Yamashiro et al. (2000) investigate the genetic composition of *L.lugubris* from a wide range including Ryukyu, Taito, and Ogasawara islands. In addition to concluding the Ryukyu geckos as invasive populations, they further proposed several ways to distinguish between invasive and native populations of *L.lugubris* by means of karyotype and molecular methods. These guide lines could be applied to study the origin of Taiwanese population in the future.

Another species, for which most local biologists are not yet aware of its status as an introduced species, is the brahminy blind snake (*Indotyphlopsbraminus*). This parthenogenic snake has been listed in the Global Invasive Species Database (GISD) as an invasive species except for its original habitat in India (Ota et al. 2004, Kraus 2009). GISD also defines *I.braminus* as an alien species in Taiwan. Similar to the previous geckos, the research of the species is scarce in Taiwan.

**Figure 16.**
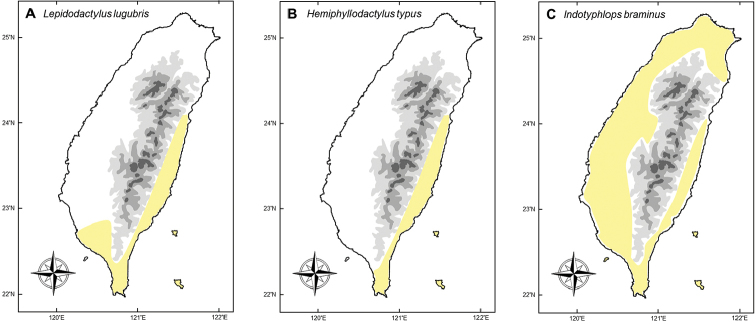
Distributions of *Lepidodactyluslugubris* (**A**) and *Hemiphyllodactylustypus* (**B**) are restricted to eastern and southern Taiwan, while *Indotyphlopsbraminus* (**C**) is believed to occupy all lowland region of the island.

Currently, the two geckos have wide distributions throughout eastern and southern Taiwan, including Orchid Island and Green Island (Figs 16A, 16B). The blind snake is distributed throughout the low land area of the entire island of Taiwan (Fig. 16C). All the three species have established large populations and would be difficult to eradicate if they are indeed invasive. The controversy of these species, with uncertain origins, is expected to be answered by more comprehensive sampling on a global scale, and with the assistance of more powerful genetic tools in the future.

### Other high-risk species

Fourteen species, including three amphibians, four lizards, two snakes, four turtles, and one crocodilian (listed in Table 2) are considered to have a high-risk of invasion into Taiwan in the future.

The cane toad, *Rhinellamarina*, might be one of the most notorious invasive anurans in the world. Established populations have spread and expanded to huge population sizes in southern Ryukyu, which is located less than 200 km from eastern Taiwan (Ota et al. 2004). Considering the similar climate, this species has a high potential to invade Taiwan. A similar situation exists for the green anole, *Anoliscarolinensis*, which has successfully invaded southern Okinawa, Ogasawara Islands, and Hawaii Islands (Kraus 2009). The third species which represent high invasion risk is *Polypedatusleucomystax*. It has successfully colonized in a wide range throughout Ryukyu Archipelago (Ota et al. 2004). Its congener, *P.megacephalus*, has invaded in Taiwan and caused serious impacts to the native *P.braueri*; while the probable colonization of a third congener might worsen the current situation. Potential invasion of these three species should be considered serious and should be monitored to avoid what could be a serious invasion.

The other species listed in Table 2 are all popular pets in the pet trade. Except for the small salamander *Cynopsorientalis*, all species in the list are medium- to large-sized reptiles. Most reported cases are giant lizards or pythons which escape to urban or suburban areas, usually due to improper housing facilities that are not secure enough to house these strong animals. Some of the turtles might be intentionally released because they grow too large to be handled. Escaped individuals of alligator snapping turtle (*Macrochelystemminckii*), common snapping turtle (*Chelydraserpentina*), Asian water monitor (*Varanussalvator*), Nile monitor (*V.niloticus*), and the common caiman (*Caimancrocodilus*) have been found in different drainages by fishermen or tourists; all are believed from human release. The reticulated python (*Malayopythonreticulatus*) has been found in rural region for several times, which has raised potential safety concerns. The latest case which caused public panic occurred on 17 January 2019, when a 4.5-m python was killed by train accident in Fangshan township, southern Taiwan. The tissue of this snake has been collected for molecular analysis, which might help to clarify the origin of this individual.

We do not consider these large reptiles to currently form invasive populations in Taiwan, but the disastrous cases of invasive reptiles in Ryukyu, Japan and Florida, USA serve as a useful reminder of the potential invasion risks and catastrophic ecological outcomes.
